# Full Coupling Modeling on Multi-Physical and Thermal–Fluid–Solid Problems in Composite Autoclave Curing Process

**DOI:** 10.3390/ma18071471

**Published:** 2025-03-26

**Authors:** Zhuoran Yang, Luohong Liu, Dinghe Li

**Affiliations:** 1 Sino-European Institute of Aviation Engineering, Civil Aviation University of China, Tianjin 300300, China; jmyang@cauc.edu.cn; 2 College of Aeronautical Engineering, Civil Aviation University of China, Tianjin 300300, China; llh6808@163.com

**Keywords:** composite laminates, autoclave curing process, full coupling modeling, multi-physical problem, thermal–fluid–solid coupling problem

## Abstract

In this study, a multi-physical and thermal–fluid–solid coupling model was developed to simulate the autoclave curing process of composite materials, aiming to explore the influence mechanism of the external flow field on the curing process. First, the extended layerwise method (XLWM) and finite volume method were adopted to simulate the composite laminates and heating airflows, respectively. Then, the thermo-chemical–mechanical-seepage analysis was carried out for the composite laminates. Considering the interaction between the airflows and laminates, a weak coupling method was proposed to solve the thermal–fluid–solid coupling problem, which consists of two parts: unidirectional coupling and bidirectional coupling. In numerical examples, the results of the two coupling schemes were compared, which indicated that the bidirectional coupling scheme consumed fewer computing resources but achieved similar accuracy.

## 1. Introduction

Composite material has been widely used in numerous domains due to its light weight, high strength, and corrosion resistance. Especially, the autoclave curing process of second-generation composite materials has played an important role in the manufacturing of aviation structures [1]. In the autoclave curing process, the heating airflows enter the autoclave from the inlet, and the composite laminates located inside are subjected to the combined action of multiple physical fields. Through thermal convection and conduction, the laminates are heated, leading to the curing reaction of the resin matrix. Then, the resin matrix transforms into a solid state, resulting in the generation of internal stresses and deformation. Meanwhile, the curing reaction further generates heat, coupled with the constraints from the mold, thereby exacerbating the internal stress and deformation of the cured laminates.

The physical fields involved in the autoclave curing process mainly include the force field, thermal field, curing degree field, and seepage field. In the authors’ previous study [2], a thermo-chemical–mechanical-seepage coupling model was developed based on the extended layerwise method (XLWM). To further improve the simulation accuracy, the interaction between the heating airflows and the composite structure needs to be considered.

Regarding the fluid–solid coupling problem, the existing solving methods can be divided into two categories: the strong coupling method and the weak coupling method. The former is to solve the fluid, solid, and fluid–solid interfaces in a system of equations, which is difficult to operate in practical applications. The latter is to call different solvers to calculate the fluid and solid domains and transmit the variations in both domains at the interface. The weak coupling method can be further divided into two schemes according to the coupling direction, namely the unidirectional coupling scheme and the bidirectional coupling scheme. The former only considers the unidirectional interaction between the fluid domain and the solid domain, while the latter considers the bidirectional interaction between both domains. This study adopted the weak coupling method to solve the thermal–fluid–solid coupling problem during the autoclave curing process of composite materials. By comparing the results of the two coupling schemes, the excellent performance of the bidirectional coupling scheme was verified. For the thermal–fluid–solid coupling problem, XLWM was adopted to solve the solid domain of composite laminates, and the finite volume method was used to solve the fluid domain of the heating airflows.

The rest of this paper is structured as follows. Section 2 reviews the state of the art of numerical simulation and experimental research on the autoclave curing process. Section 3 introduces the XLWM used to develop the solver for the solid domain. In Section 4, the solver for the fluid domain and its basic principle are described. Section 5 describes the workflow of two thermal–fluid–solid coupling schemes. The demonstration and comparison of the two coupling schemes are carried out in Section 6. The final section ends the paper with conclusions and future work.

## 2. Background and Motivation

With the increasing application of autoclave technology in composite materials, several studies are devoted to the numerical simulation and experiment of the curing process [3,4] and the cured composite structures [5,6,7]. For the moving mesh technique, Greco et al. [8] and Funari et al. [9] studied the related problems of crack growth in combination with the multiscale technique and fracture mechanics, respectively. To investigate the influence of heating airflows on the composite structures, the fluid–solid coupling method [10] should be employed. Regarding the porosity in the curing process, Torres et al. [11] studied the mechanism of pore formation through X-ray computed tomography. It was found that the space between adjacent layers greatly affected the final porosity. Hudson et al. [12] used ultrasonic detection to detect and locate pores in the cured composite materials. Simacek et al. [13] simulated the curing process of thermosetting composites. Three possible outcomes after cross-deformation and extrusion flow were predicted, including the infiltration of fiber/matrix suspension and resin into pores, and spread deformation. Dei et al. [14] proposed a multi-physical analysis model to predict the pore generation in cured composite structures. Šimáček et al. [15] demonstrated the modeling of pore-filling and reinforcement compression processes under pressure and confirmed the practicality of the model. In addition, Sandeep et al. [16] proposed a new in situ experimental method to measure the dimensional change of composite laminates during the curing process. Ogugua et al. [17] estimated the energy consumption of an autoclave curing process of composite materials based on two models. The first model was established based on the simplified heat capacity equation. The second model combined the Multi-Relaxation-Time Lattice Boltzmann method with the Fourier heat equation. In addition, the physical information neural networks were employed to solve the governing equation of the coupling problem and simulate the thermo-chemical evolution during the composite curing process [18].

Regarding the autoclave curing process, the influence of internal and external conditions on cured composite structures was investigated from various perspectives. For instance, Netzel et al. [19] investigated the influence of angular radius and 0° fiber ratio on corner defects. Moretti et al. [20] investigated the effect of adhesive on the deformation of cured structures. Netzel et al. [21] studied the impact of hygroscopicity on the curing process, thereby affecting the mechanical properties [22] and thermal properties [23] of cured composites. Pereira et al. [24] investigated the property changes of L-shaped composite structures, which considered the influence of the external environment. Ali et al. [25] studied the effect of fiber type and autoclave-treatment temperature on the mechanical and interfacial properties of fiber-reinforced 3D printed composites. Pourahmadi et al. [26] investigated the interlaminar shear strength and other properties of carbon/polyether ether ketone composite laminates.

The fluid–solid coupling is a classic engineering problem that has been continuously studied to develop high-precision solution methods [27]. Marcela et al. [28] calculated the submerged flexible solids in an interleaved way over time and proved that the separator could reduce the hydrodynamic coefficient on the separator cylinder. Zhang et al. [29] proposed an interfacial coupling method to solve the fluid–solid coupling problem with large deformation. Abdolhossein et al. [30] studied the natural frequency variation of liquid-filled cylindrical shells in a fluid–solid coupled thermal environment. Kohlstädt et al. [31] investigated the fluid–solid coupling problem during high-pressure die casting and calculated the deformation of test parts. Han et al. [32] designed a novel marine composite blade based on the fluid–solid coupling method. Huang et al. [33] explored the characteristic parameters of composite propellers under fluid–solid coupling conditions.

The existing research on the autoclave curing process mainly focused on solving multi-physical fields in the solid domain. The mainstream method was to simulate the heating airflows acting on the composite structure through the unidirectional effect of airflows on solids. However, the two-way scheme of fluid–solid coupling was rarely applied in the curing process. Therefore, in this study, two fluid–solid coupling schemes were proposed for the curing process, namely the unidirectional coupling scheme and the bidirectional coupling scheme. The interaction between fluid and solid domains was sufficiently considered in both schemes.

## 3. Solver of the Solid Domain: Extended Layerwise Method

### 3.1. Mixed Variational Principle of Thermo-Chemical–Mechanical-Seepage Coupling

**Figure 1:**
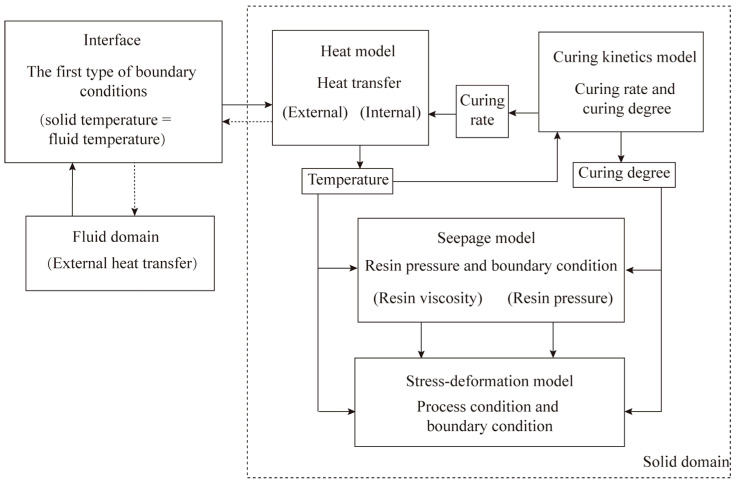
Coupling of multi-physical fields in autoclave forming process.

The production of composite structures in an autoclave is a complex process that involves multiple physical fields simultaneously, as shown in Figure 1. Based on the Law of Conservation of Momentum, the Law of Conservation of Energy, Darcy’s Law, and the dynamic relationship of curing reaction measured by experiment [34], the thermo-chemical–mechanical-seepage governing equations are as follows:

(1) $$\begin{array}{l} \sigma_{ij,j}+f_{i}=\rho {\ddot{u}}_{i}\\ h_{i,i}-s=-\Theta^{0}\dot{\eta }\\ v_{i,i}-\dot{p}=0\\ \dot{c}=g\left(T,c\right) \end{array},i,j=1,2,3$$

where $\sigma_{ij}$, $h_{i}$ and $\nu_{i}$ are the symmetric Cauchy stress tensor, heat flux vector, and Darcy velocity, respectively. c is the curing degree. p is the pore pressure. $f_{i}$ and s represent volume force and heat source, respectively. η, $\Theta^{0}$, and ρ are entropy density, constant positive reference temperature, and density, respectively. g(T,c) is the function of temperature T and degree of curing c based on curing kinetics.

The mixed variational principle equivalent to Equation (1) is as follows

(2) $$\begin{array}{l} \int_{V}\sigma_{\alpha \beta }\delta \varepsilon_{\alpha \beta }dV+\int_{V}\rho \frac{\partial^{2}u_{\alpha }}{\partial t^{2}}dV-\int_{V}\delta u_{\alpha }f_{\alpha }dV-\int_{\Gamma_{F}}\delta u_{\alpha }F_{\alpha }d\Gamma \\ -\int_{V}h_{\alpha }\delta e_{\alpha }dV-\int_{V}\delta \theta (\Theta^{0}\dot{\eta }-s)dV-\int_{\Gamma }\hbar \delta \theta d\Gamma \\ -\int_{V}v_{i}\delta m_{i}dV+\int_{V}\delta p\dot{p}dV-\int_{\Gamma }V\delta pd\Gamma \\ -\int_{V}g(T,c)\delta cdV+\int_{V}\dot{c}\delta cdV=0 \end{array}$$

where α=1,2,3 represents the displacement components in the *x*, *y*, and *z* directions, respectively. θ is the temperature change. $\varepsilon_{\alpha \beta }$ and $e_{\alpha }$ are the strain and the temperature gradient, respectively. ℏ is the heat flow on the boundary. $m_{i}$ is the pore pressure field vector. The four rows in the above equation represent the energy changes caused by the mechanical field, temperature field, seepage field, and curing field, respectively.

The proposed multi-physical boundary conditions are as follows:

(3) $$\begin{array}{l} \sigma_{ij}n_{j}=F_{i}\;\;\mathrm{on}\;\Gamma_{F},u_{i}={\bar{u}}_{i}\;\mathrm{on}\;\Gamma_{u}\\ h_{i}n_{i}=\hbar \;\mathrm{on}\;\Gamma_{H},\theta =\bar{\theta }\;\mathrm{on}\;\Gamma_{\theta }\\ v_{i}n_{i}=V\;\mathrm{on}\;\Gamma_{V},p=\bar{p}\;\mathrm{on}\;\Gamma_{p} \end{array}$$

where $u_{i}$ denotes the displacement component. $n_{i}$ denotes the component of the unit external normal vector. $\Gamma_{F}$, $\Gamma_{u}$, $\Gamma_{H}$, $\Gamma_{\theta }$, $\Gamma_{V}$, and $\Gamma_{p}$ are the boundaries of the analysis region of force, displacement, heat flux, temperature, Darcy velocity, and pore pressure, respectively.

### 3.2. XLWM for Thermo-Chemical–Mechanical-Seepage Coupling

To simulate the possible damage caused by the thermo-chemical–mechanical-seepage multi-physical loading, XLWM was used to model the cured composite laminates. The delamination damage was considered inherent. In XLWM, the multi-physical variations at point (*x*, *y*, *z*) are discrete along the thickness direction, which are expressed as

(4) $$\begin{array}{l} u_{\alpha }(x,y,z,t)=\sum_{k=1}^{N+2}\phi_{k}(z)u_{\alpha ik}(x,y,t)+\sum_{k=1}^{N_{D}}\Xi_{k}(z)u_{\alpha lk}(x,y,t)+\sum_{k=2}^{N+1}\Theta_{k}(z)u_{\alpha rk}(x,y,t)\\ \theta (x,y,z,t)=\sum_{k=1}^{N+2}\phi_{k}\left(z\right)\theta_{ik}(x,y,t)+\sum_{k=1}^{N_{D}}\Xi_{k}(z)\theta_{lk}(x,y,t)+\sum_{k=2}^{N+1}\Theta (z)\theta_{rk}(x,y,t)\\ c(x,y,z,t)=\sum_{k=1}^{N+2}\phi \left(z\right)c_{ik}(x,y,t)+\sum_{k=1}^{N_{D}}\Xi_{k}(z)c_{lk}(x,y,t)\\ p(x,y,z,t)=\sum_{k=1}^{N+2}\phi_{k}\left(z\right)p_{ik}(x,y,t)+\sum_{k=1}^{N_{D}}\Xi_{k}(z)p_{lk}(x,y,t)+\sum_{k=2}^{N+1}\Theta_{k}(z)p_{rk}(x,y,t) \end{array}$$

where $u_{\alpha ik}$, $u_{\alpha lk}$, and $u_{\alpha rk}$ are the displacement freedom of interpolation points, the additional freedom of displacement discontinuity caused by delamination damage, and the additional freedom of strain discontinuity caused by the interface, respectively. $\theta_{ik}$, $\theta_{lk}$, and $\theta_{rk}$ are the temperature freedom of interpolation points, the additional freedom of temperature discontinuity caused by delamination damage, and the additional freedom of temperature gradient discontinuity caused by the interface, respectively. $c_{ik}$ and $c_{lk}$ are the curing degree freedom of interpolation points and the discontinuity curing degree freedom caused by delamination damage, respectively. $p_{ik}$, $p_{lk}$, and $p_{rk}$ are the pore pressure freedom at interpolation points, the additional freedom of pore pressure discontinuity caused by delamination damage, and the additional freedom of pore pressure gradient discontinuity caused by the interface, respectively. *N* is the layer number of composite laminates. The standard and additional freedom are, respectively, N+2 and N. $N_{D}$ denotes the number of nodes expanded by delamination. $\phi_{k}(z)$, $\Xi_{k}\left(z\right)$, and $\Theta_{k}(z)$ are the corresponding shape function used to describe the strong continuous displacement field, weak continuous stress field, and strong discontinuity in a composite laminate with lamination damage.

The gradient relationships of strain, temperature, and pore pressure are expressed as

(5) $$\begin{array}{l} \varepsilon_{kl}=\frac{1}{2}(u_{k,l}+u_{l,k})\\ e_{k}=-\theta_{,k}\\ m_{k}=-p_{,k} \end{array}k,l=1,2,3$$

where $\varepsilon_{kl}$, $e_{k}$, θ, and $m_{k}$ are the strain tensor, thermal field vector, pore pressure vector, and temperature change, respectively.

The constitutive equations of thermo-chemical–mechanical-seepage coupling are

(6) $$\begin{array}{l} \sigma_{ij}=C_{ijkl}\varepsilon_{kl}-\lambda_{ij}\theta -\gamma_{ij}c\\ \eta =\lambda_{kl}\varepsilon_{kl}+\varrho \theta \\ h_{i}=\kappa_{ij}e_{j}\\ v_{i}=k_{ij}m_{i} \end{array}$$

where $C_{ijkl}$, ϱ and $\lambda_{ij}$ are the stiffness coefficient, thermal expansion coefficient, and thermal stress-temperature constant, respectively. Note that $\varrho =\rho_{r}c/\vartheta$, where c is the specific heat per unit mass at a constant volume, and $\rho_{r}$ is the density of resin.

Referring to Equation (1), the multi-physical variations are discrete along the thickness direction and thus need to be expressed by the in-plane elements. The Lagrange interpolations for the node displacements of each in-plane element are

(7) $$\begin{array}{l} u_{\alpha \zeta k}(x,y,t)=\psi_{m}(x,y){\tilde{U}}_{\alpha \zeta km}(t)\\ \theta_{\zeta k}(x,y,t)=\psi_{m}(x,y){\tilde{\theta }}_{\zeta km}(t)\\ p_{\zeta k}(x,y,t)=\psi_{m}(x,y){\tilde{p}}_{\zeta km}(t)\\ c_{\zeta k}(x,y,t)=\psi_{m}(x,y){\tilde{c}}_{\zeta km}(t) \end{array}$$

According to Equations (1)–(7), the finite element (FE) equations of thermo-chemical–mechanical-seepage XLWM are obtained. Details can be found in the authors’ previous work [2]. The final FE governing equation is expressed as

(8) $$\left[\begin{array}{cccc} M^{uu} & 0 & 0 & 0\\ 0 & 0 & 0 & 0\\ 0 & 0 & 0 & 0\\ 0 & 0 & 0 & 0 \end{array}\right]\left\{\begin{array}{c} \ddot{U}\\ 0\\ 0\\ 0 \end{array}\right\}+\left[\begin{array}{cccc} 0 & 0 & 0 & 0\\ C^{\theta u} & C^{\theta \theta } & C^{\theta c} & 0\\ 0 & 0 & C^{pp} & 0\\ 0 & 0 & 0 & C^{cc} \end{array}\right]\left\{\begin{array}{c} \dot{U}\\ \dot{\Theta }\\ \dot{P}\\ \dot{C} \end{array}\right\}+\left[\begin{array}{cccc} K^{uu} & K^{u\theta } & K^{uc} & 0\\ 0 & K^{\theta \theta } & 0 & 0\\ 0 & 0 & K^{pp} & 0\\ 0 & 0 & 0 & 0 \end{array}\right]\left\{\begin{array}{c} U\\ \Theta \\ P\\ C \end{array}\right\}=\left\{\begin{array}{c} F^{u}\\ 0\\ 0\\ F^{c} \end{array}\right\}$$

### 3.3. Newmark-Based Time Integration of XLWM

For the thermo-chemical–mechanical-seepage coupling problems, the mixed time integration algorithm was employed to solve the multi-physical responses in the governing equation (i.e., Equation (8)). In the proposed mixed integration algorithm, the elastic response was obtained by the Newmark method, while the temperature change, pore pressure and curing degree were obtained by the Crank–Nicolson method. The multi-physical variations and derivatives at time t+∆t are approximated in terms of their values as time *t*, which are expressed as

(9) $$\begin{array}{l} P_{t+△t}=P_{t}+[(1-\beta_{1}){\dot{P}}_{t}+\beta_{1}{\dot{P}}_{t+△t}]△t\\ C_{t+△t}=C_{t}+[(1-\beta_{1}){\dot{C}}_{t}+\beta_{1}{\dot{C}}_{t+△t}]△t\\ \Theta_{t+△t}=\Theta_{t}+[(1-\beta_{1}){\dot{\Theta }}_{t}+\beta_{1}{\dot{\Theta }}_{t+△t}]△t\\ {\dot{U}}_{t+△t}={\dot{U}}_{t}+[(1-\gamma ){\overset{..}{U}}_{t}+\gamma {\overset{..}{U}}_{t+△t}]△t\\ U_{t+△t}=U_{t}+U_{t}△t+[(\frac{1}{2}-\beta_{2}){\overset{..}{U}}_{t}+\beta_{2}{\overset{..}{U}}_{t+△t}]△t^{2} \end{array}$$

Based on the proposed mixed-time integration algorithm, the final integration equation of Equation (8) is obtained as

(10) $$\hat{K}Q_{t+△t}={\hat{F}}_{t+△t}$$

where

(11) $$Q_{t+△t}={\left[U_{t+△t},\Theta_{t+△t},C_{t+△t},P_{t+△t}\right]}^{T}$$

(12) $$\hat{K}=\left[\begin{array}{cccc} c_{0}M^{uu}+K^{uu} & K^{u\theta } & K^{uc} & 0\\ c_{1}C^{\theta u} & c_{8}C^{\theta \theta }+K^{\theta \theta } & C^{\theta c}c_{8} & 0\\ 0 & 0 & c_{8}C^{pp}+K^{pp} & 0\\ 0 & 0 & 0 & c_{8}C^{cc} \end{array}\right]$$

(13) $${\hat{F}}_{t+△t}=\left\{\begin{array}{c} F_{t+△t}^{u}+M^{uu}(c_{0}U_{t}+c_{2}{\dot{U}}_{t}+c_{3}{\overset{..}{U}}_{t})\\ C^{\theta u}(c_{1}{\dot{U}}_{t}+c_{4}{\dot{U}}_{t}+c_{5}{\overset{..}{U}}_{t})+C^{\theta c}(c_{8}C_{t}+c_{9}{\dot{C}}_{t})+C^{\theta \theta }(c_{8}\Theta_{t}+c_{9}{\dot{\Theta }}_{t})\\ C^{pp}c_{8}P_{t}+C^{pp}c_{9}{\dot{P}}_{t}\\ \int_{-H/2}^{H/2}g\left(\Theta_{t+△t},c_{t+△t}\right)\Phi_{\zeta k}dz+C^{cc}(c_{8}C_{t}+c_{9}{\dot{C}}_{t}) \end{array}\right\}$$

## 4. Solver of the Fluid Domain: The Finite Volume Method

The simulation of the airflow field was mainly based on Stanford University’s Unstructured Grid Navier–Stokes code, also called SU2 (v7.5.1, Stanford University, Stanford, CA, USA). As an open-source computational fluid dynamics (CFD) suite, SU2 is used to calculate various compressible and incompressible fluids by the finite volume method. The governing equation of airflows is as follows [35]:

(14) $$\frac{\partial U}{\partial t}+\nabla \cdot F^{c}-\nabla \cdot (\mu^{\nu k}F^{\nu k})=Q$$

where k=1, 2. U is the vector of state variables. $F^{c}$ is the convective flux. $F^{vk}$ is the viscous flux. Q is a generic source term. ∇⋅(⋅) represents the divergence operator. $\mu^{v1}$ is the total viscosity as a sum of dynamic and turbulent components. $\mu^{v2}$ is the effective thermal conductivity.

The Reynolds number of the fluid (i.e., Re) is calculated by the following formula to determine the state of the fluid:

(15) $$Re=\frac{\rho vd}{\mu }$$

where ρ, v, d, and μ are the air density, velocity, characteristic length (i.e., autoclave diameter), and dynamic viscosity of airflows, respectively. Note that when Re > 8000, the fluid is turbulent; when Re < 2300, the fluid is laminar; otherwise, the fluid is transitional.

## 5. Thermo–Fluid–Solid Coupling Method

The existing research on the composite autoclave curing process mainly considers the influence of the fluid domain on the solid domain. To further improve the simulation accuracy, the interaction between the solid and fluid domains is supposed to be considered. Therefore, a weak coupling method was adopted to solve the thermal–fluid–solid coupling problem in this paper. The unidirectional and bidirectional coupling schemes were both employed. The existing methods are equivalent to the unidirectional coupling scheme. On this basis, the bidirectional coupling scheme was proposed, which sufficiently considered the interaction between the solid and fluid domains. In the unidirectional coupling scheme, the values of multi-physical variations were transferred from fluid to solid in a time step, while these values interacted between fluid and solid in the bidirectional coupling scheme.

### 5.1. Unidirectional Coupling Scheme

**Figure 2:**
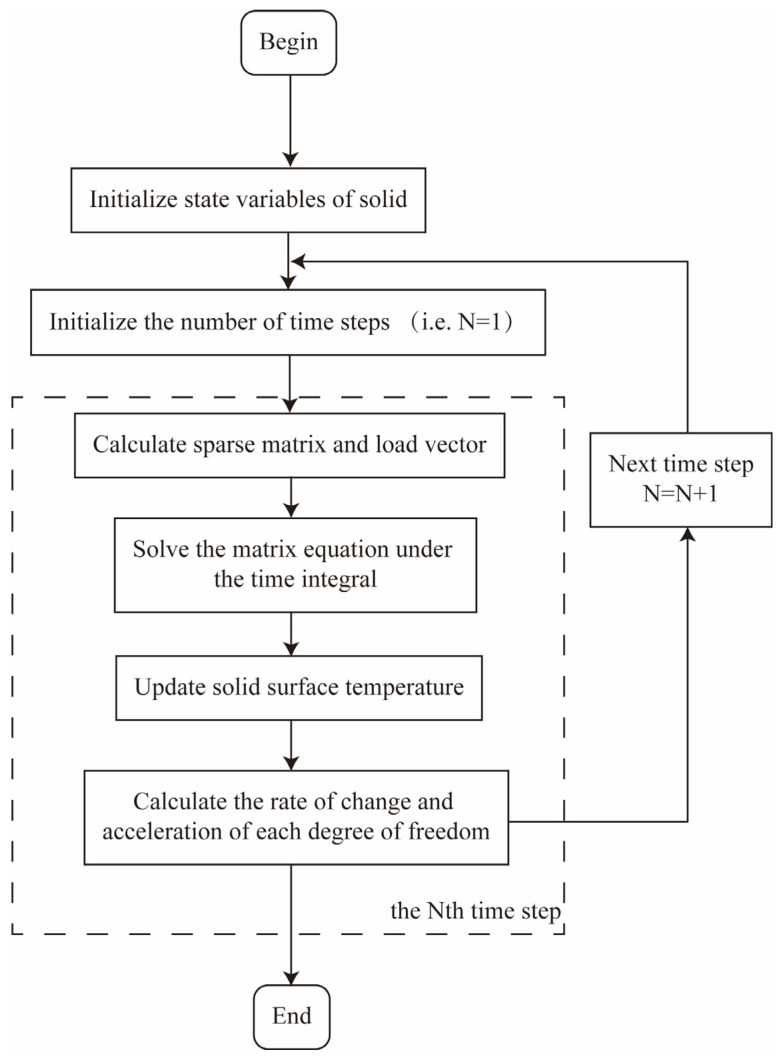
Flow chart of solving unidirectional coupled solid domain.

In the unidirectional coupling scheme, only the influence of the fluid domain on the solid domain was considered. The interface temperature was calculated in the fluid domain and then transmitted to the solid domain. As shown in Figure 2, at the beginning of the Nth time step (i.e., at time $t_{n-1}$), the solvers of the fluid and solid domains are simultaneously activated. At the end of the Nth time step (i.e., at time $t_{n}$), the results obtained from the fluid domain are regarded as the boundary conditions of the solid domain in the next time step.

**Figure 3:**
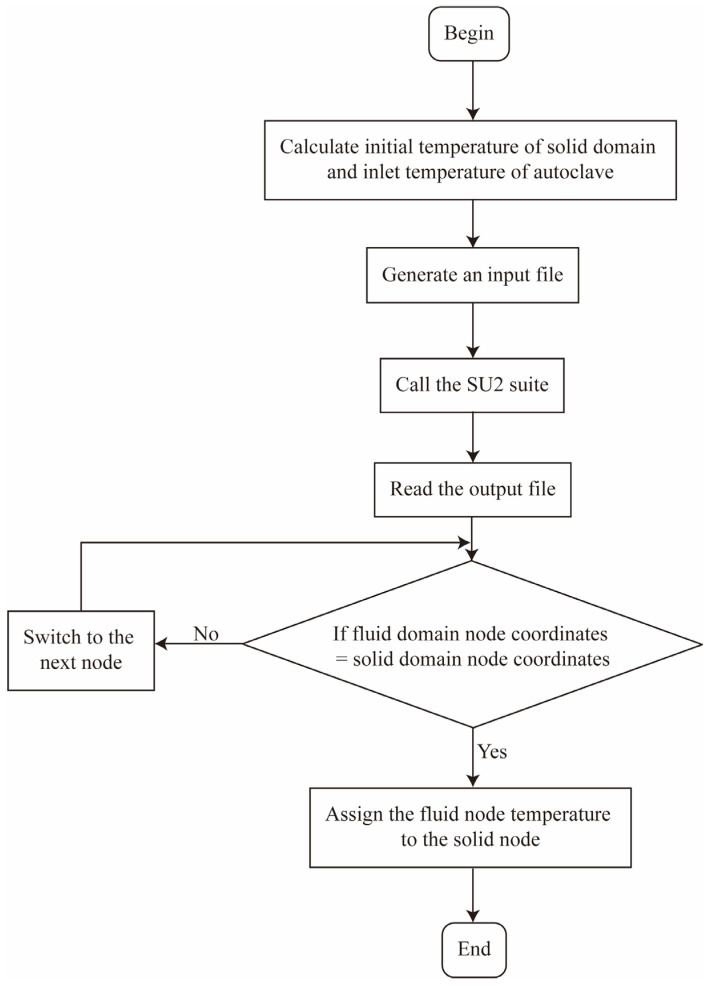
Flow chart of value passing at the fluid-solid interface.

The first type of boundary condition, i.e., the solid temperature at the interface is equal to the fluid temperature, was considered for the data transmission from the fluid domain to the solid domain at the interface. Figure 3 presents the flow chart for updating the boundary temperature of the solid domain, including the following steps:

1. Calculate the initial temperature of the solid domain and the inlet temperature of the autoclave, and adjust the relevant initial settings (e.g., the inlet speed, fluid density, etc.). Then, an input file is generated for the open-source suite (i.e., SU2);
2. Call SU2 to calculate the fluid domain and save results to the output file;
3. Read the output file, and define the corresponding temperature as the solid boundary based on the node coordinates;
4. According to the nodal coordinates in the fluid domain, search for the corresponding interface node, and assign the temperature value to each node of the solid domain.

### 5.2. Bidirectional Coupling Scheme

**Figure 4:**
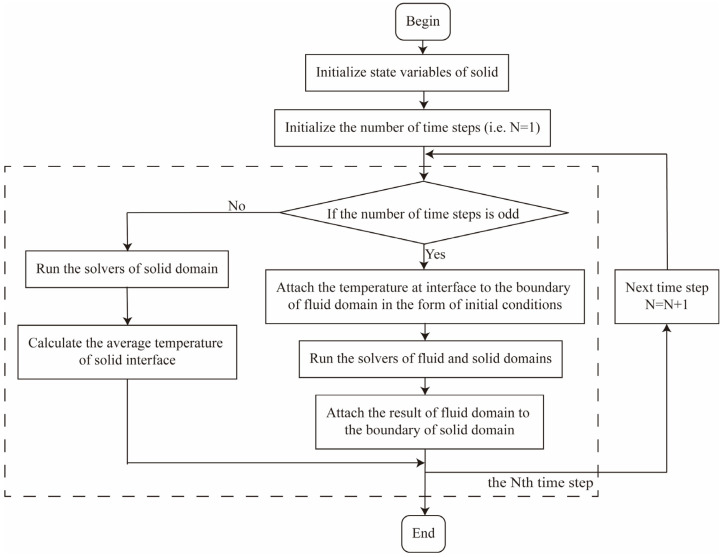
Flow chart of solving solid domain in bidirectional coupling scheme.

The interaction between the fluid domain and the solid domain was considered in the bidirectional coupling scheme. Figure 4 presents the workflow of solving solid domain in the bidirectional coupling scheme. Taking the Nth time step (i.e., at time $t_{n-1}$) as an example, the calculation process includes the following steps.

If N is odd, then

1. At the beginning of the Nth time step (i.e., at time $t_{n-1}$), define the temperature at the interface as the boundary of the fluid domain through the solid domain as the initial condition;
2. Start the solvers of the fluid and solid domains simultaneously;
3. At the end of the Nth time step (i.e., at time $t_{n}$), terminate the calculation in the fluid domain, and define the result of the fluid domain as the boundary of the solid domain.

If N is even, then

1. Start the solvers of the solid domains;
2. At the end of the Nth time step (i.e., at time $t_{n}$), calculate and define the average temperature of the solid interface as one of the initial conditions in the fluid domain for the next time step.

The steps for transferring values in the fluid–solid domain are the same as those in the unidirectional coupling scheme. However, the values transferred in the solid–liquid domain require calculating the temperature at the fluid–solid interface. To simplify the calculation, the average temperature of the solid interface was set as the initial temperature of the fluid interface. After calculation, the initial temperature of the fluid interface in the next time step was obtained according to the first type of boundary conditions.

## 6. Numerical Examples

### 6.1. Curing Simulation of a Carbon Fiber/Epoxy Resin Laminated Plate

**Figure 5:**
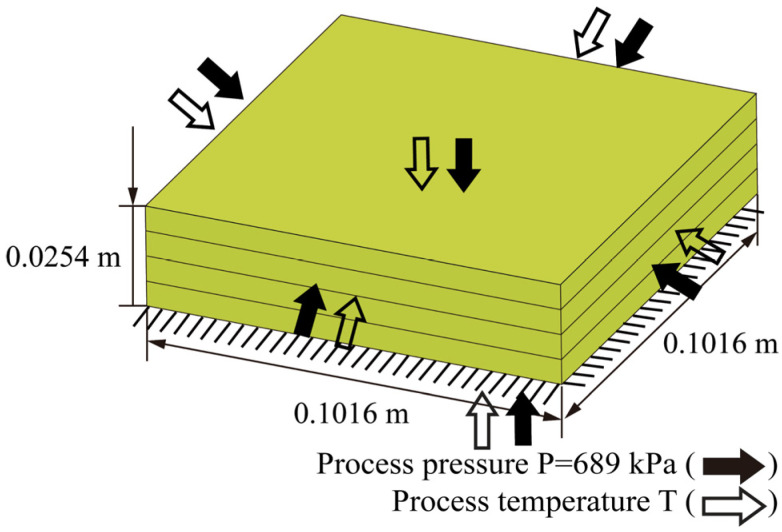
Geometry of the carbon fiber/epoxy resin laminated plate (AS4/3501-6).

A carbon fiber/epoxy resin laminated plate was employed to determine the rationality of the bidirectional coupling method. The geometric dimensions of the laminated plate are 0.1016 m×0.1016 m×0.0254 m. The stacking sequence was set to 0 °/ 90 °/90 °/0 °. As shown in Figure 5, the bottom of the plate was completely fixed, and the top surface and four sides were subjected to a uniform pressure P=689 kPa. The initial temperature was set to 273.15 K. The material properties of the resin are listed in Table 1 [36].

**Figure 6:**
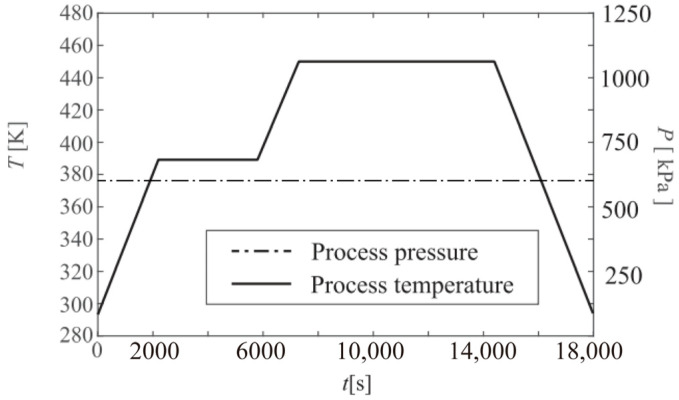
Inlet temperature and outlet pressure curve.

**Figure 7:**
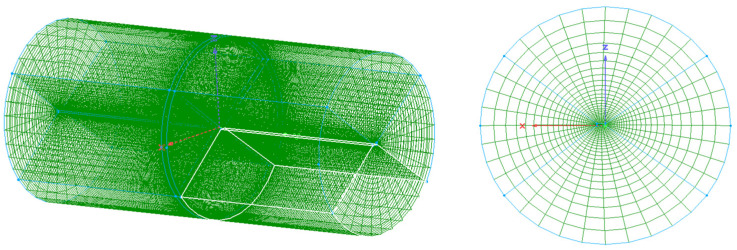
Meshing of the airflow field around the laminated plate in an autoclave.

The carbon fiber/epoxy resin laminated plate was placed in the center of a transverse horizontal cylinder autoclave with a diameter of 3 m and a height of 6 m. The initial conditions of the fluid domain are shown in Table 2. The heating of airflows to the solid was controlled by the temperature of the autoclave inlet. Figure 6 shows the temperature curve of the inlet and the pressure curve of the outlet [36]. For the convenience of data transmission, the meshing of the fluid domain and solid domain at the interface was the same. The remaining parts of the fluid and solid domains were meshed according to the same proportion to ensure the convergence, as shown in Figure 7. The blue and green lines in the figure are the boundaries and gridlines of the fluid domain. To better compare the fully coupled model (i.e., bidirectional coupling scheme) with the previous method, the previous method is simulated as unidirectional coupling scheme, the two schemes are calculated under the same conditions, and the results are compared. The same research method will be adopted for the subsequent examples.

**Figure 8:**
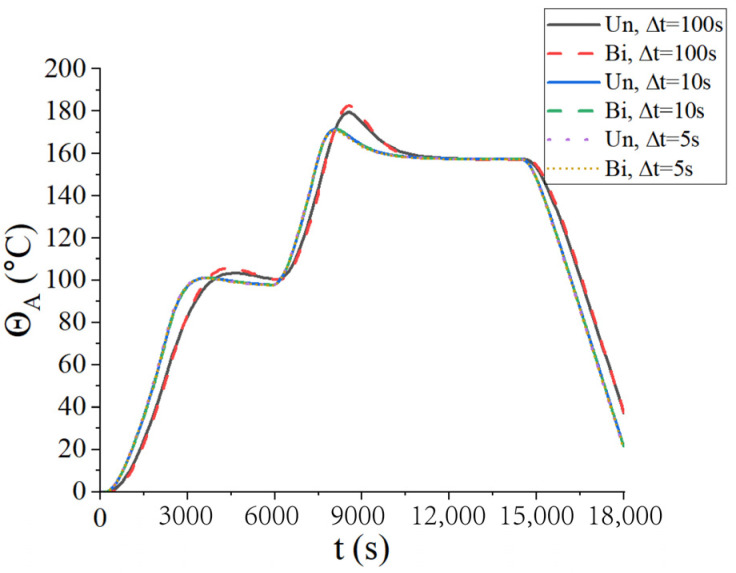
Central temperatures of the laminated plate for three different time steps.

Firstly, the convergence of the time step was considered. Three types of time steps were selected for two coupling schemes: ∆*t* = 100 s, ∆*t* = 10 s, and ∆*t* = 5 s. The central temperature change curves under three kinds of time steps are shown in Figure 8, where Un represents the unidirectional coupling and Bi represents the bidirectional coupling. It is found that the temperature curves are convergent at ∆t=10 s. To reduce the computational load, the time step ∆*t* = 10 s was adopted in the following examples.

**Figure 9:**
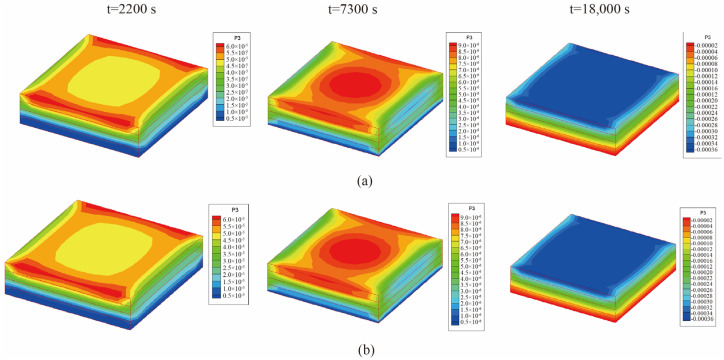
Displacement (*u*_3_) of the laminated plate when ∆t = 10 s (unit: m): (**a**) unidirectional coupling scheme; (**b**) bidirectional coupling scheme.

From Figure 8, it is observed that the temperature of the center point of the laminated plate rose gradually with the heating of the airflows. After the heating stage, the temperature rose until it slightly exceeded the temperature of the airflows, due to the heat released by the internal curing reaction. In the heat preservation process, the heat generated from the curing process was transferred to the airflows; thus, the temperature of the laminated plate gradually approached the airflow temperature. Figure 9 presents the displacement (*u*_3_) of the laminated plate at the end of the first heating (i.e., *t* = 2200 s), second heating (i.e., *t* = 7300 s), and heat release (i.e., *t* = 18,000 s). The central displacement after the first heating was slightly lower than that of the sides, while in the second heating stage, the displacements of the center and sides were almost equal. This is due to the heat preservation after the first heating. When the entire heating process was completed, the laminated plate was completely cured; thus, the deformation tended to be uniform. Besides, it is found from Figure 8 and Figure 9 that the calculation results of the unidirectional coupling scheme and the bidirectional coupling scheme are consistent for the time step of 10 s.

**Figure 10:**
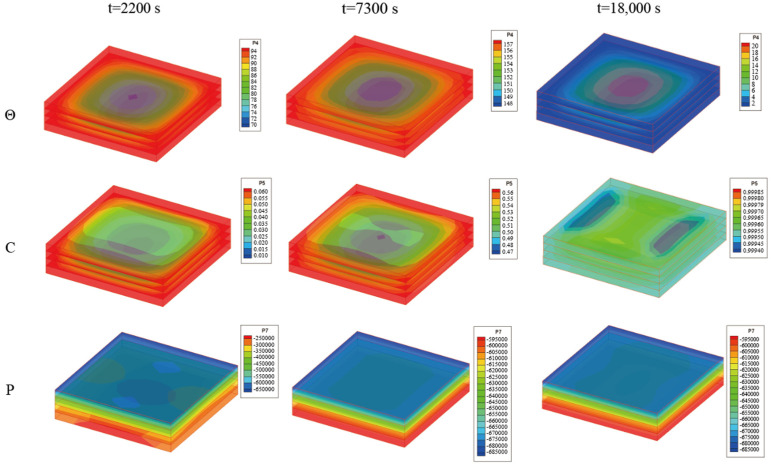
Temperature, curing degree, and pore pressure (i.e., Θ, C, and P) of the laminated plate obtained by bidirectional coupling scheme when ∆t = 10 s (unit: °C, 1, Pa).

**Figure 11:**
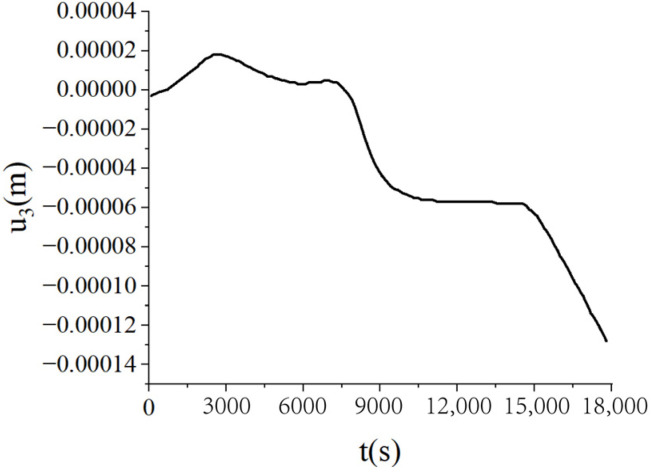
Central displacement (*u*_3_) of the laminated plate by the bidirectional coupling scheme when ∆t = 10 s (unit: m).

Figure 10 presents the temperature, curing degree, and pore pressure of the laminated plate obtained by the bidirectional coupling scheme. At the beginning of the curing process, the plate mainly relied on external heating, leading to a lower central temperature and curing degree. As the curing process was completed, the overall curing degree of the plate was close to 1. The central temperature was higher than the side temperature due to the incomplete heat transfer from internal curing to the outside. Figure 11 presents the change curve of the central displacement of the laminates obtained by the bidirectional coupling scheme. As can be seen from the figure, in the two heating processes, the central displacement increases due to thermal expansion, while in the beginning stage of the insulation process, the central displacement decreases due to curing and seepage. In the heat dissipation process, the central displacement also gradually decreases due to heat release.

### 6.2. Curing Simulation of a Carbon Fiber/Epoxy Resin Plate with Mold

**Figure 12:**
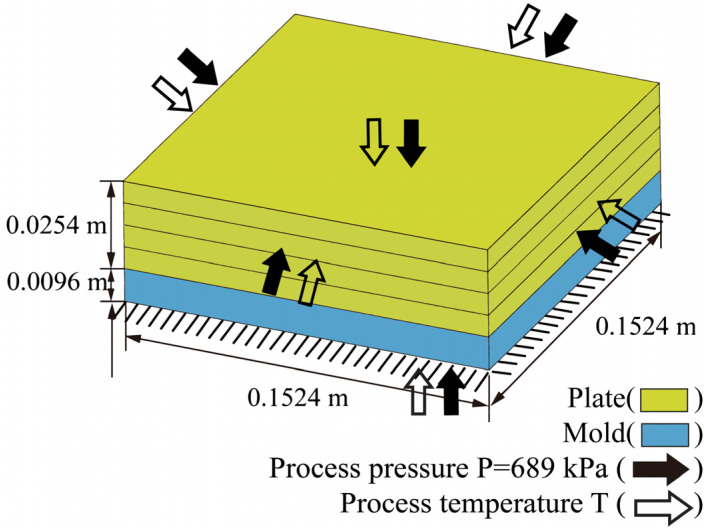
Geometry of the carbon fiber/epoxy resin laminated plate (AS4/3501-6) with mold.

In the real curing process, the mold always has a significant impact on the heat transfer. In this example, a mold was designed and attached under the carbon fiber/epoxy resin laminated plate, as shown in Figure 12. The geometric dimensions of the mold are 0.1016 m×0.1016 m×0.0096 m, and the thermodynamic parameters are listed in Table 3. The dimensions, material parameters, and boundary conditions of the laminated plate are the same as those in the previous example.

**Figure 13:**
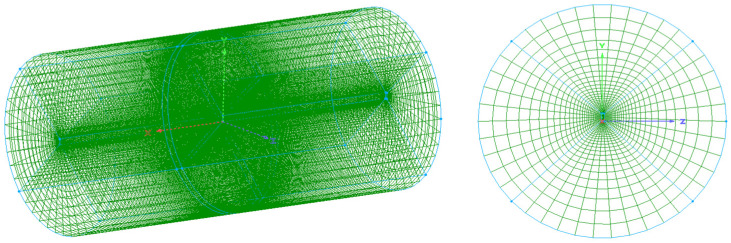
Meshing of the airflow field around the laminated plate with mold in an autoclave.

**Figure 14:**
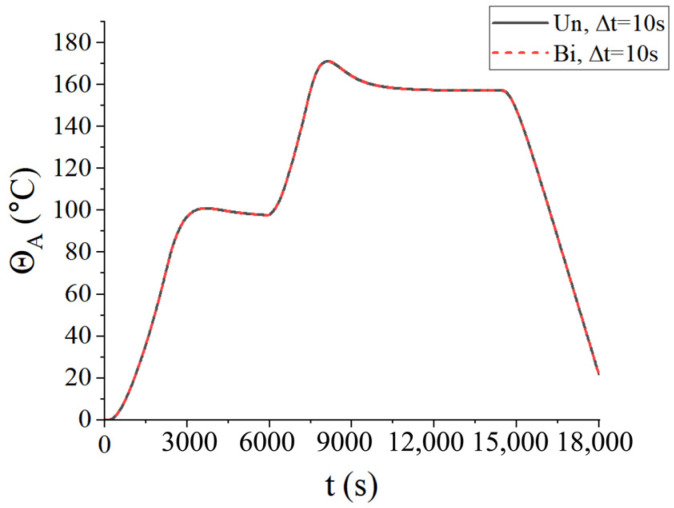
Central temperatures of the laminated plate with mold when ∆t = 10 s.

The mold and the carbon fiber/epoxy resin laminated plate were placed in the center of the autoclave. The initial condition of the fluid domain remained constant, as shown in Table 2. The single-layer mesh was used in the mold, and the meshing scheme at the fluid–solid interface was the same as in the previous example. To ensure convergence in the fluid domain, the meshing of the remaining parts was kept in the corresponding proportions. The meshing of the fluid domain is shown in Figure 13. The blue and green lines in the figure are the boundaries and gridlines of the fluid do-main. The central temperature curves of the laminated plate with mold are shown in Figure 14.

**Figure 15:**
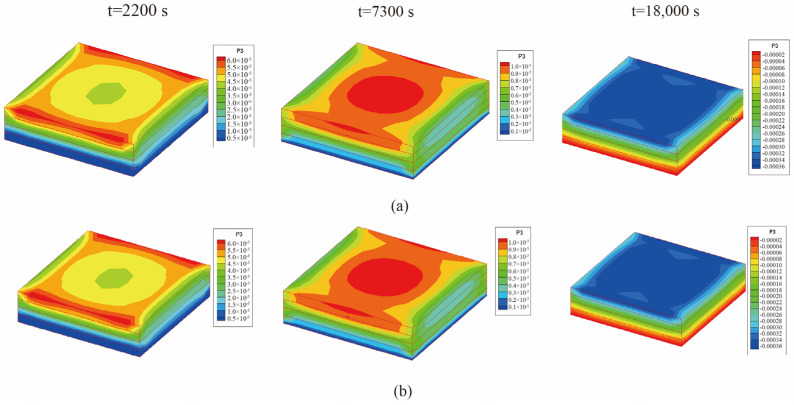
Z-axis displacement of the laminated plate with mold when ∆t = 10 s (unit: m): (**a**) unidirectional coupling scheme; (**b**) bidirectional coupling scheme.

Figure 15 presents the displacement (*u*_3_) of the laminated plate with mold at the end of the first heating (i.e., t = 2200 s), second heating (i.e., t = 7300 s), and heat release (i.e., t = 18,000 s). According to Figure 14 and Figure 15, it is observed that the central temperature and displacement distributions are basically the same as in the previous example. Due to the difference in thermal conductivity between the mold and the laminated plate, the curing rate slowed down in the early stage of the heating process, and thus the displacement distribution was different from that in the previous example. In addition, by comparing the results for the time step of 10 s, it is found that the temperature and displacement distributions obtained using the two coupling schemes are identical.

### 6.3. Curing Simulation of a Curved Carbon Fiber/Epoxy Resin Plate

**Figure 16:**
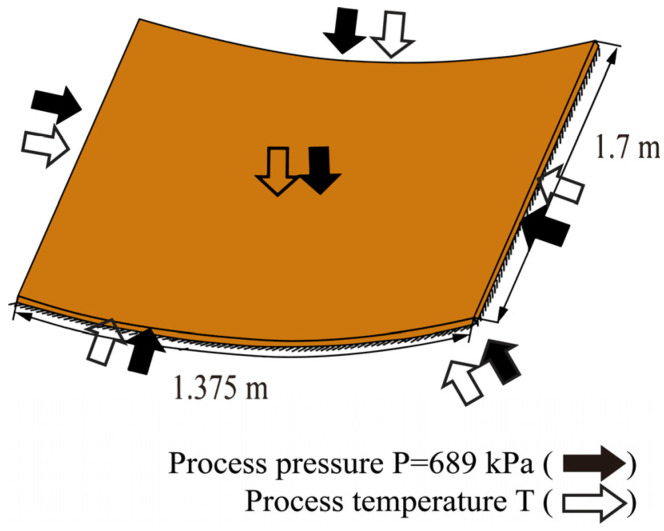
Geometric dimensions of the curved carbon fiber/epoxy resin laminated plate (AS4/3501-6).

In this example, the curing simulation of the curved carbon fiber/epoxy resin plate was carried out. The geometric dimensions are shown in Figure 16. The bottom arc length of the cross-section is 1.375 m and the height is 1.7 m. The material parameters are the same as those in the first example, as listed in Table 1. For the curved plate, it is necessary to introduce the transformation relation matrix of the local coordinate system and global coordinate system [37].

**Figure 17:**
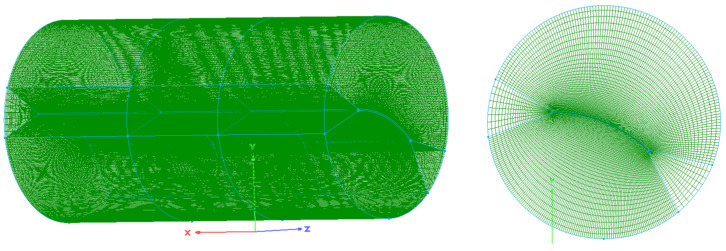
Meshing of the airflow domain around the curved plate.

**Figure 18:**
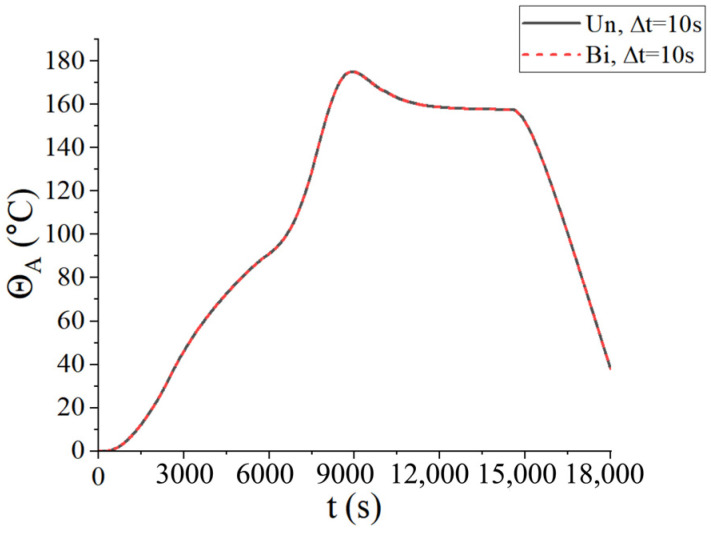
Central temperatures of the curved plate when ∆t = 10 s.

The curved plate was placed in the center of the autoclave. The initial condition of the airflow is shown in Table 2. Since the curved plate is large, the meshing of the smooth part needs to be further refined to ensure convergence, as shown in Figure 17. The blue and green lines in the figure are the boundaries and gridlines of the fluid do-main. For the time step of 10 s, the central temperatures of the curved plate are shown in Figure 18.

**Figure 19:**
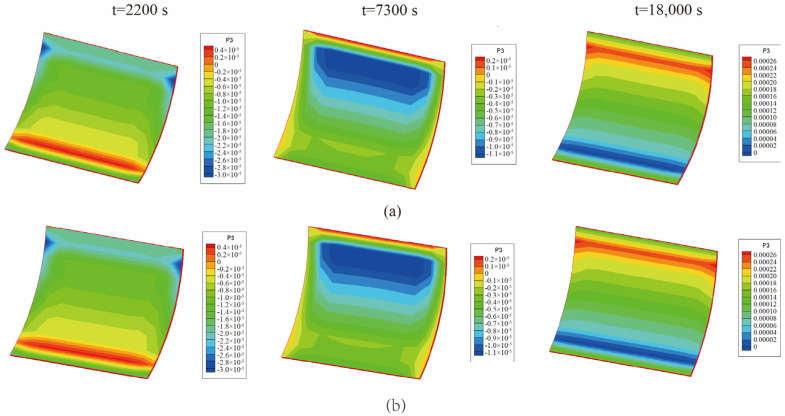
Z-axis displacement of the curved plate when ∆t = 10 s (unit: m): (**a**) unidirectional coupling scheme; (**b**) bidirectional coupling scheme.

Figure 19 presents the displacement (*u*_3_) of the curved plate at the end of the first heating (i.e., *t* = 2200 s), second heating (i.e., *t* = 7300 s), and heat release (i.e., *t* = 18,000 s). Due to the larger contact area between the curved plate and the airflow, the heat transfer between the curved plate and the fluid domain was relatively uniform, resulting in a gradient distribution of displacement in the normal direction. In addition, it can be seen from Figure 18 and Figure 19 that the results obtained by the two coupling schemes are identical.

### 6.4. Curing Simulation of a Carbon Fiber/Epoxy Resin Plate with Delamination

**Figure 20:**
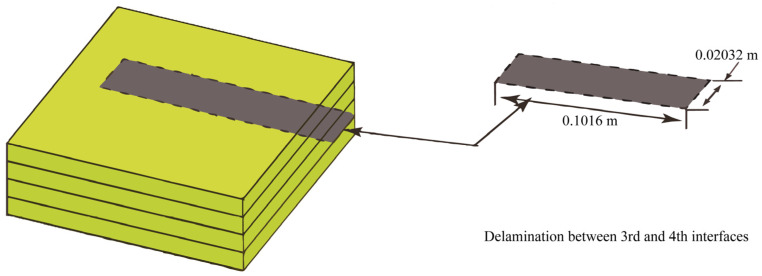
Geometry of the carbon fiber/epoxy resin laminated plate (AS4/3501-6) with delamination.

**Figure 21:**
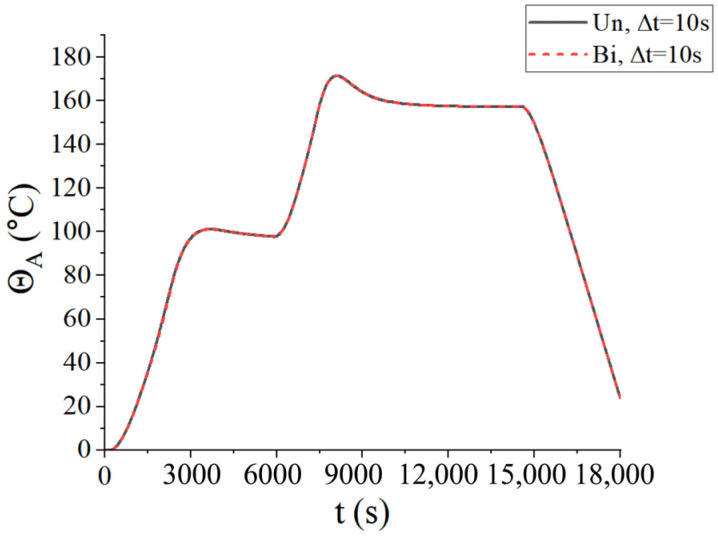
Central temperatures of the laminated plate with delamination when ∆t = 10 s.

Defects in composite materials often occur during the curing process. In this example, the curing of a laminated plate with delamination was simulated. As shown in Figure 20, a delaminated area of 0.1016 m×0.02032 m was created inside the plate. The dimensions, material parameters, and boundary conditions of the plate are the same as those in the first example. The initial condition and the meshing of the fluid domain remained constant. The central temperature curves of the laminated plate with delamination are shown in Figure 21.

**Figure 22:**
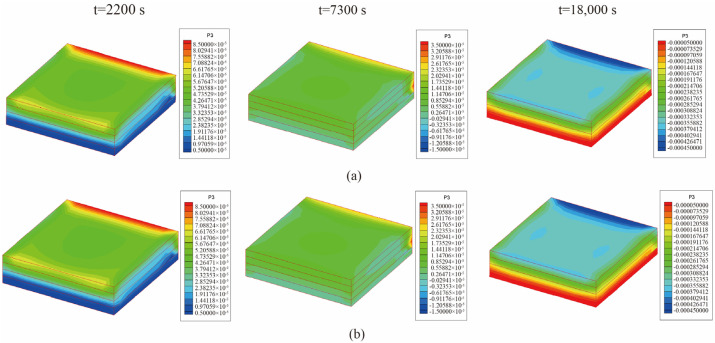
Z-axis displacement of the laminated plate with delamination when ∆t = 10 s (unit: m): (**a**) unidirectional coupling scheme; (**b**) bidirectional coupling scheme.

Figure 22 presents the displacement (u3) of the laminated plate at the end of the first heating (i.e., t = 2200 s), second heating (i.e., t = 7300 s), and heat release (i.e., t = 18,000 s). Due to the delamination on one side of the plate, there is a significant difference in displacement between the top and bottom of the delamination defect and other areas. In addition, the results obtained by the two coupling schemes are identical, as shown in Figure 21 and Figure 22. However, it is worth noting that in all the previous examples, the state of the fluid domain obtained using the bidirectional coupling scheme was calculated within half of the time step, which consumed less computation time and cost.

## 7. Conclusions

In this study, a full coupling analysis model of multi-physical fields and thermal–fluid–solid was established, which considered the influence of the internal airflows on the composite curing process in an autoclave. Both unidirectional coupling and bidirectional coupling schemes were adopted to solve the thermal–fluid–solid coupling problem. Unidirectional coupling was used to simulate the previous research methods, and bidirectional coupling was a fully coupled model. By comparing the results of both schemes, it was concluded that the bidirectional coupling scheme has higher computational efficiency and effectively saves computational costs. The calculation results showed that the displacements of the center and sides were identical due to the effect of the heat preservation process, while the temperature gap was caused by different heat transfer distances. Moreover, the delamination phenomenon significantly amplified the displacement differences at the corresponding positions.

Future work will concentrate on simulating structural delamination and crack damage during the autoclave curing process, and further solve geometric nonlinear problems of composite materials.

## Figures and Tables

**Table 1:** Material parameters of AS4/3501-6.

Physical Quantity	Symbol	Value
Density	ρ	$1790\;kg/m^{3}$
Specific Heat Capacity	$C_{p}$	862 J/(kg⋅K)
Young’s Modulus	E	$E_{11}=105.02\;GPa,\;E_{22}=E_{33}=105.02\;GPa$
Thermal Conductivity	k	$k_{11}=12.83\;W/(m\cdot K),\;k_{22}=k_{33}=0.4135\;W/(m\cdot K)$
Coefficient of Thermal Expansion	α	$5.76\times 10^{-5}\;K^{-1}$
Shear Modulus	G	$G_{23}=2.4945\;GPa,\;G_{12}=G_{13}=3.1942\;GPa$
Poisson’s Ratio	ν	$\nu_{12}=\nu_{13}=0.2686$ $,\;\nu_{23}=0.34531$

**Table 2:** Initial conditions of fluid domain.

Physical Quantity	Symbol	Value
Outlet Pressure	$P_{b}$	600 kPa
Density	ρ	$1.225\;kg/m^{3}$
Viscosity Coefficient	μ	$1.7894\times 10^{-5}\;kg/(m\cdot s)$
Velocity of Flow	v	2.5 m/s
Prandtl Number	Pr	0.72

**Table 3:** Material parameters of the mold.

Physical Quantity	Symbol	Value
Density	ρ	$2700\;kg/m^{3}$
Specific Heat Capacity	$C_{p}$	615.9 J/(kg⋅K)
Young’s Modulus	E	69 GPa
Thermal Conductivity	k	$1.96\times 10^{5}\;W/(m\cdot K)$
Coefficient of Thermal Expansion	α	$1.7\times 10^{-5}\;K^{-1}$
Poisson’s Ratio	ν	0.35

## Data Availability

The original contributions presented in this study are included in the article. Further inquiries can be directed to the corresponding author.

## Nomenclature

$\sigma_{ij}$	Symmetric Cauchy stress tensor
$h_{i}$	Heat flux vector
$\nu_{i}$	Darcy velocity
c	Curing degree
p	Pore pressure
$f_{i}$	Volume force
s	Heat source
η	Entropy density
$\Theta^{0}$	Constant positive reference temperature
ρ	Density
g(T,c)	The function of temperature T and degree of curing c based on curing kinetics
α=1,2,3	Displacement components in the *x*, *y,* and *z* directions
θ	Temperature change
$\varepsilon_{\alpha \beta }$	Strain gradient
$e_{\alpha }$	Temperature gradient
ℏ	Heat flow
$m_{i}$	Pore pressure field vector
$u_{i}$	Displacement component
$n_{i}$	Component of the unit external normal vector
$\Gamma_{F}$	The boundaries of the analysis region of force
$\Gamma_{u}$	The boundaries of the analysis region of displacement
$\Gamma_{H}$	The boundaries of the analysis region of heat flux
$\Gamma_{\theta }$	The boundaries of the analysis region of temperature
$\Gamma_{V}$	The boundaries of the analysis region of Darcy velocity
$\Gamma_{p}$	The boundaries of the analysis region of pore pressure
$u_{\alpha ik}$	Displacement freedom of interpolation points
$u_{\alpha lk}$	Additional freedom of displacement discontinuity caused by delamination damage
$u_{\alpha rk}$	Additional freedom of strain discontinuity caused by the interface
$\theta_{ik}$	Temperature freedom of interpolation points
$\theta_{lk}$	Additional freedom of temperature discontinuity caused by delamination damage
$\theta_{rk}$	Additional freedom of temperature gradient discontinuity caused by the interface
$c_{ik}$	Curing degree freedom of interpolation points
$c_{lk}$	Discontinuity curing degree freedom caused by delamination damage
$p_{ik}$	Pore pressure freedom at interpolation points
$p_{lk}$	Additional freedom of pore pressure discontinuity caused by delamination damage
$p_{rk}$	Additional freedom of pore pressure gradient discontinuity caused by the interface
N	The layer number of composite laminates
$N_{D}$	The number of nodes expanded by delamination
$\phi_{k}(z)$	The corresponding shape function used to describe the strong continuous displacement field in a composite laminate with lamination damage
$\Xi_{k}\left(z\right)\;$	The corresponding shape function used to describe the weak continuous stress field in a composite laminate with lamination damage
$\Theta_{k}(z)$	The corresponding shape function used to describe the strong discontinuity in a composite laminate with lamination damage
$\varepsilon_{kl}$	Strain tensor
$e_{k}$	Thermal field vector
$m_{k}$	Pore pressure vector
$C_{ijkl}$	Stiffness coefficient
ϱ	Thermal expansion coefficient
$\lambda_{ij}$	Thermal stress-temperature constant
c	Specific heat per unit mass at a constant volume
$\rho_{r}$	Density of resin
U	Vector of state variables
$F^{c}$	Convective flux
$F^{vk}$	Viscous flux
Q	Generic source term
∇⋅(⋅)	Divergence operator
$\mu^{v1}$	Total viscosity as a sum of dynamic and turbulent components
$\mu^{v2}$	Effective thermal conductivity
Re	The Reynolds number of fluid
ρ	Air density
v	Velocity
d	Characteristic length
μ	Dynamic viscosity of airflows
